# Ischemia with Nonobstructive Coronary Artery Disease and Atrial Cardiomyopathy—Two Sides of the Same Story?

**DOI:** 10.3390/life13020443

**Published:** 2023-02-04

**Authors:** Irina Afrăsânie, Iulian Theodor Matei, Sabina Andreea Leancă, Adriana Chetran, Alexandru Dan Costache, Vlad-Adrian Afrăsânie, Bianca-Ana Dmour, Daniela Crișu, Minerva Codruța Bădescu, Lăcrămioara Ionela Șerban, Irina Iuliana Costache

**Affiliations:** 1 Cardiology Clinic, “St. Spiridon” County Clinical Emergency Hospital, 700111 Iași, Romania; 2 Department of Internal Medicine, “Grigore T. Popa” University of Medicine and Pharmacy, 700115 Iași, Romania; 3 Department of Cardiovascular Rehabilitation, Clinical Rehabilitation Hospital, 700661 Iași, Romania; 4 Department of Medical Oncology, “Grigore T. Popa” University of Medicine and Pharmacy, 700115 Iași, Romania; 5 Department of Oncology, The Regional Institute of Oncology, 700483 Iași, Romania; 6 Internal Medicine Clinic, “St. Spiridon” County Clinical Emergency Hospital, 700111 Iași, Romania; 7 Department of Physiology, “Grigore T. Popa” University of Medicine and Pharmacy, 700115 Iași, Romania

**Keywords:** INOCA, coronary microvascular dysfunction, atrial cardiomyopathy, atrial fibrillation, heart failure, endothelial dysfunction, systemic inflammation, comorbidities

## Abstract

Ischemia with nonobstructive coronary artery disease (INOCA) is increasingly recognized as a significant cause of angina, myocardial remodeling, and eventually heart failure (HF). Coronary microvascular dysfunction (CMD) is a major endotype of INOCA, and it is caused by structural and functional alterations of the coronary microcirculation. At the same time, atrial cardiomyopathy (ACM) defined by structural, functional, and electrical atrial remodeling has a major clinical impact due to its manifestations: atrial fibrillation (AF), atrial thrombosis, stroke, and HF symptoms. Both these pathologies share similar risk factors and have a high comorbidity burden. CMD causing INOCA and ACM frequently coexist. Thus, questions arise whether there is a potential link between these pathologies. Does CMD promote AF or the reverse? Which are the mechanisms that ultimately lead to CMD and ACM? Are both part of a systemic disease characterized by endothelial dysfunction? Lastly, which are the therapeutic strategies that can target endothelial dysfunction and improve the prognosis of patients with CMD and ACM? This review aims to address these questions by analyzing the existing body of evidence, offering further insight into the mechanisms of CMD and ACM, and discussing potential therapeutic strategies.

## 1. Introduction

Ischemic heart disease and atrial fibrillation (AF) are major cardiovascular diseases that cause a significant burden on the health system, with increased morbidity and mortality, not only in the elderly but also among the younger population. While obstructive coronary artery disease (CAD) has been extensively characterized with well-defined therapeutic options, ischemia with nonobstructive coronary artery disease (INOCA) still faces many unanswered questions regarding pathophysiology and optimal therapy, despite being increasingly recognized as a significant cause of angina, myocardial remodeling, and eventually heart failure (HF) [1]. Up to 70% of the patients with angina and proven ischemia referred for invasive coronary angiography have nonobstructive CAD, reflecting the high burden of this pathology [2]. INOCA encompasses two major endotypes: epicardial coronary vasospasm and coronary microvascular dysfunction (CMD) caused by structural and functional alterations of the coronary microcirculation [2]. The consequences of INOCA are not only anginal symptoms but also manifestations of heart failure (HF) due to subsequent alterations in ventricular function. CMD is a frequent encounter in patients with HF with preserved ejection fraction (HFpEF), for which it has been proposed as a potential mechanism [3].

At the same time, AF is more than just an arrhythmia, as it might reflect an underlying diseased atrium with structural, functional, and electrical remodeling, all of these processes being incorporated under the term ‘atrial cardiomyopathy’ (ACM). ACM has been recently recognized as a clinically relevant entity in which atrial dysfunction is considered the primordial pathological alteration, leading to cardiovascular consequences such as AF, HFpEF, and embolic stroke due to atrial thrombosis even in the absence of atrial arrhythmias [4].

Despite apparently different cardiovascular pathogenies, coronary vascular dysfunction and ACM share similar risk factors, such as arterial hypertension, diabetes mellitus (DM), obesity, obstructive sleep apnea (OSA), or dysfunctional epicardial adipose tissue (EAT) [4,5]. Moreover, CMD and AF are often found in association and are both contributors to HFpEF development [3]. Therefore, questions arise about whether CMD and ACM share similar pathophysiological pathways. It has been proposed that CMD and AF are part of a systemic vascular disease, characterized by endothelial dysfunction (ED) leading to alterations in the microvasculature of various organs, including the heart [6]. The hypothesis of increased comorbidity burden causing systemic inflammation and ED could explain the coexistence of CMD and ACM in this category of patients [7].

There are limited data exploring the potential link between these pathologies, whether they share similar pathogenic mechanisms or whether one promotes the other. Additionally, the particular association between ACM and CMD has been poorly characterized in previously published papers. This review aims to describe existing evidence on the relationship between ACM and phenotypes of INOCA with a focus on CMD. We also explore the role of systemic ED as an underlying pathogeny and describe its associations with atrial ED and CMD. In the second part of the manuscript, current knowledge of potential therapeutic strategies targeting the common pathogenic substrate of these diseases is addressed. We emphasize the role of non-pharmacological measures such as weight loss and continuous positive airway pressure (CPAP) therapy in OSA and question whether EAT could be a new therapeutic target to improve cardiovascular outcomes. Finally, we discuss the available evidence on pharmacotherapies with a potential role in modulating systemic ED, ACM manifestations, and CMD.

## 2. Definitions and Characterization

### 2.1. Anatomy of the Coronary Circulation and INOCA Endotypes

The coronary arterial system includes three major compartments, each with a specific vessel structure and function. The proximal compartment is formed by the epicardial coronary arteries, which are large, conductive vessels with a three-layer wall and a main role in transportation [1,8]. Coronary microcirculation includes arteries with a diameter smaller than 500 µm, and it is the main site of flow and vascular resistance modulation. It comprises the intermediate compartment formed of pre-arterioles and the distal compartment including arterioles and capillaries [9]. Arterioles modulate up to 55% of the coronary microvascular resistance by adjusting their vascular tone in response to metabolites produced by the surrounding cardiomyocytes [9].

CMD encompasses structural and functional abnormalities of the coronary microcirculation that alter the myocardial perfusion and ultimately cause ischemia. Structural remodeling implies luminal narrowing of arterioles by media thickening and intimal proliferation, as well as perivascular fibrosis and capillary rarefaction [10]. Functional abnormalities include impaired vasodilatation and microvascular spasm. Altered vasodilatation has endothelium-dependent mechanisms, in the context of endothelial dysfunction, due to decreased production of nitric oxide (NO), as well as endothelium-independent pathways that are less well described and might be related to impaired relaxation of vascular smooth muscle cells [1]. Epicardial vasospasm is the second endotype of INOCA, and it can be caused by hyperreactivity of vascular smooth muscle cells or endothelial dysfunction with decreased NO production and paradoxical vasoconstriction [2].

Currently, there are no available instruments to directly visualize the coronary microvasculature. The diagnostic algorithm of INOCA has been already extensively described [2]. In brief, CMD can be assessed using noninvasive techniques such as positron emission tomography (PET) or cardiac magnetic resonance (CMR), which measure the coronary flow reserve (CFR) as the ratio of hyperemic blood flow in response to vasoactive agents, such as adenosine or regadenoson, divided by resting blood flow. These methods evaluate only the endothelium-independent vasodilatation. Another noninvasive method is transthoracic Doppler echocardiography with the measurement of CFR. However, the gold standard for the diagnosis of INOCA endotypes requires invasive coronary functional testing with the measurement of CFR and index of microvascular resistance (IMR) after adenosine infusion. Abnormal values indicative of CMD are less than 2 for CFR and greater than 25 for IMR, respectively. This first step assesses endothelial-independent CMD, and it should be followed by vasoreactivity testing with intracoronary acetylcholine (ACh), which evaluates endothelial-dependent coronary dysfunction and can identify either isolated epicardial vasospasm, microvascular spasm, or both [2].

### 2.2. Endothelial Dysfunction

Although composed of a single row of cells covering the endocardium and the blood vessels, the endothelium plays a major role in the modulation of vascular tone and contributes to processes such as inflammation, hemostasis, or thrombosis. Endothelial cells synthesize NO in response to hemodynamic shear stress, which promotes vasodilatation and inhibits endothelial thrombosis [11]. In addition, they produce molecules such as Von Willebrand factor (VWF) and other circulating microparticles which mediate platelet activation and aggregation, leading to thrombus formation. Endothelial cells express on their surface adhesion molecules which recruit immune cells, promoting vascular inflammation in adjacent tissues. When the endothelium becomes dysfunctional, due to structural loss of integrity or functional dysregulation, altered vasodilatation or paradoxical vasoconstriction as well as a prothrombotic state occur, causing various clinical manifestations [12]. Multiple studies have previously shown a strong association between ED and traditional cardiovascular risk factors such as obesity, DM, OSA, and arterial hypertension [13]. Moreover, systemic ED has been associated with increased risk of major adverse cardiovascular events (MACE), even in patients without typical risk factors [14]. Existing studies used different methods to describe ED, such as circulating biomarkers (VWF, NO), histological evaluation of tissue fragments, or methods that evaluate the vasodilatory response of the peripheral vascular endothelium [15]. The latter offer an indirect measure of systemic endothelial function, and the most widely used techniques are flow-mediated vasodilatation (FMD) of the brachial artery and reactive hyperemia index (RHI) quantified by peripheral arterial tonometry (PAT). Brachial FMD assesses echographically the ability of the brachial artery to dilate after an increase in blood flow caused by forearm suprasystolic occlusion with a pressure cuff, as a result of NO synthesis in response to increased shear stress, and it is a marker of the endothelial-dependent function of conduit arteries [15]. RHI-PAT is derived from measuring finger pulse amplitude changes caused by a hyperemic surge after a period of ischemia induced with an occlusive cuff placed around the arm, and it is indicative of microvascular ED [16].

### 2.3. Atrial Cardiomyopathy

The first detailed analysis of ACM was published in 2016, in an Expert Consensus Document of several societies, which defined it as “any complex of structural, architectural, contractile or electrophysiological changes affecting the atria with the potential to produce clinically relevant manifestations”. The pathophysiological processes leading to the development of ACM are frequently intricate and include atrial cardiomyocyte dysfunction, fibrotic changes, and non-fibrotic atrial infiltration with inflammatory cells or adipocytes [4]. These alterations further induce electrical atrial remodeling with the development of atrial arrhythmias. AF is a clinical manifestation of ACM, and it is frequently used in published work as a surrogate for ACM. Beyond altered electrophysiological properties, ACM associates atrial structural remodeling reflected in atrial dilatation and atrial mechanical dysfunction with decreased atrial compliance and atrial contractile capacity [17]. All of these changes adversely impact ventricular filling, leading to manifestations of HF, and ACM is a frequent cause of HFpEF [18]. At the same time, atrial alterations lead to an atrial prothrombotic state, even in the absence of atrial arrhythmias, and ACM may be in the causal pathway of cardioembolic stroke independently of AF [19]. There is no standard diagnosis for ACM, and published studies use heterogeneous methods to define ACM, which are mainly related to electrocardiographic abnormalities of the P wave, the presence of AF, abnormal atrial electrophysiologic (EP) properties on EP studies, abnormal atrial histological features, and increased atrial dimensions or atrial dysfunction on different imaging techniques such as echocardiography or CMR [20].

## 3. Risk Factors

Both CMD and ACM share similar risk factors, suggesting a possible common pathophysiological pathway causing these pathogenies. Traditional cardiovascular risk factors and emerging entities with deleterious cardiovascular effects such as OSA and dysfunctional EAT promote ED, which contributes to CMD and ACM development, reinforcing their possible association [4,5].

### 3.1. Traditional Cardiovascular Risk Factors

Aging is associated with significant atrial remodeling, due to increased atrial fibrosis. Atrial dilatation is more severe in the elderly, and AF incidence increases with age [4]. Moreover, increasing age was found to be associated with CMD and epicardial vasospasm as a consequence of abnormal coronary function induced by vascular senescence [21].

Smoking significantly increases the risk of AF by up to 33%, and it is also associated with INOCA endotypes [22,23]. The negative effect of smoking is initiated by induced ED. Smoking compounds decrease endothelial NO synthesis and induce endothelial cell activation, further causing platelet adhesion and inflammatory cell recruitment. In addition, smoking increases the production of reactive oxygen species (ROS) which directly cause endothelial cell disruption and apoptosis [24].

Obesity is a comorbidity with major deleterious systemic effects. Adipose tissue is an active metabolic organ that secretes proinflammatory cytokines, creating a low-grade systemic inflammatory environment. Chronic vascular inflammation promotes ED, which alters the coronary arterial vasomotor response, causing CMD [25]. In addition, atrial ED and inflammation in the atrial coronary microcirculation promote atrial structural and electrical remodeling and induce an atrial prothrombotic state, leading to ACM manifestations [4]. Left atrium (LA) size increases proportionally to body surface area and obesity significantly augments the risk of developing AF [22,26].

Arterial hypertension is the underlying cause of at least 1 in 5 cases of incident AF, being the most prevalent modifiable risk factor for AF [22]. Increased blood pressure leads to progressive dilatation and mechanical dysfunction of the LA, and it promotes the development of an atrial arrhythmic substrate. Renin–angiotensin–aldosterone system (RAAS) activation signals proinflammatory and profibrotic pathways which cause atrial myocyte hypertrophy and atrial collagen deposition, maintaining the adverse atrial remodeling process [4]. At the same time, RAAS activation causes vascular remodeling, being a major contributor to CMD development. Angiotensin II triggers smooth muscle cell proliferation and hypertrophy, which decreases the vascular arterial lumen and can potentially lead to coronary capillary rarefaction. Moreover, angiotensin II mediates vascular inflammation and ED, contributing to coronary functional dysregulation due to decreased levels of vasodilators and abnormal response to vasoconstrictor stimuli [27].

Diabetes mellitus is another major risk factor for both CMD and ACM. Persistent hyperglycemic stress causes mitochondrial dysfunction with increased oxidative stress production, and activation of the advanced glycation end product (AGE)–AGE receptor (RAGE) system [4]. These molecular abnormalities signal profibrotic pathways in the atria, alter impulse conduction, and increase susceptibility for AF development, with a 35% higher risk of AF occurrence in diabetic patients compared to the healthy population [22]. Similarly, high oxidative levels and activation of the AGE–RAGE interaction reduce NO synthesis and cause systemic endothelial damage, including in the coronary microvasculature. Consequently, CMD is a frequent encounter in diabetic patients, and it includes both structural and functional microvascular alterations. In addition to anginal symptoms, coronary ED also mediates myocardial inflammation and fibrosis, leading to ventricular dysfunction [3].

### 3.2. Emerging Cardiovascular Risk Factors

#### 3.2.1. Obstructive Sleep Apnea

OSA is a sleep disorder characterized by multiple nocturnal pauses in breathing due to airway collapse, followed by reoxygenation. OSA is associated with multiple cardiovascular consequences, and ED appears to mediate this pathogenic link [28]. After the apneic episode resumes, reoxygenation is accompanied by a strong sympathetic drive, which impairs the endothelial function, with increased systemic inflammation and oxidative stress, reduced bioavailability of NO, and activation of prothrombotic pathways [29]. The link between OSA and systemic ED was evidenced by several studies showing impaired values of brachial FMD in patients with OSA, which occur even in the absence of other risk factors for ED such as hypertension or DM [28].

In addition, it appears that OSA might contribute to the development of CMD in patients with nonobstructive CAD, most probably due to repeated episodes of hypoxemia–reoxygenation, which resemble the ischemia–reperfusion injury. In line with this, a meta-analysis showed that OSA was significantly associated with lower CFR [30]. Another study demonstrated that OSA was present in more than half of the included patients with microvascular angina and normal epicardial coronary arteries; in addition, severe OSA was associated with significantly lower values of CFR [31].

At the atrial level, OSA triggers a true myopathic process, with electrophysiological changes caused by intermittent hypoxemia and hypercapnia, increases in intrathoracic pressure, and sympathovagal activation, which significantly augment the risk of AF [29]. In fact, in The Sleep Heart Health Study, patients with OSA had a prevalence of nocturnal AF of 4.8%, compared to 0.9% in patients without OSA [32]. Similarly, OSA has a high prevalence in AF patients, with reported rates from 21% to 74%, and it increases the risk of AF recurrence after arrhythmia ablation and cardioversion [33,34]. Ultimately, the proarrhythmic changes are accompanied by structural remodeling, characterized by atrial enlargement and dysfunction, which subsequently contribute to ventricular diastolic impairment and manifestations of HF [35].

#### 3.2.2. Epicardial Adipose Tissue—A New Cardiovascular Risk Factor?

EAT is the visceral fat deposit of the heart and it is located beneath the epicardium and in direct contact with the myocardium. It is typically located in the atrioventricular and interventricular grooves, and it can surround the entire myocardial surface when the amount of fat increases. It is also arranged along the epicardial coronary branches, and it can extend into the myocardium as it follows the coronary adventitia. There is no anatomical structure separating the myocardium from the EAT; thereby, these structures share the same microcirculation, which enables cellular and molecular interactions at this level [36].

EAT is an organ with a higher metabolic activity compared to subcutaneous fat, and under abnormal conditions, it directly exerts multiple cardiac deleterious effects. Metabolic disorders such as obesity and DM induce systemic inflammation which signals epicardial adipocyte proliferation and dysfunction. Subsequently, defective adipocytes secrete proinflammatory cytokines such as IL-6, IL-1β, and IL-8 which promote myocardial inflammation, cardiomyocyte dysfunction, and fibrosis, by paracrine and endocrine signaling [37]. All these processes lead to a metabolic-inflammatory phenotype of ventricular myopathy, characterized by reduced ventricular distensibility, leading to HFpEF [3]. In addition, increased EAT local production of vasoactive compounds such as angiotensinogen modulates coronary vascular tone and acts in conjunction with the increased inflammatory reaction to promote functional CMD, capillary rarefaction, and atherogenesis [36,38]. Indeed, it has been demonstrated that pericoronary adipose tissue is correlated with the development and severity of coronary atherosclerotic plaques, which suggests that EAT might play a role in the initiation and progression of coronary atherosclerosis, possibly through induced coronary ED and sustained inflammation [39]. Moreover, several studies showed that in patients with INOCA, EAT thickness is independently associated with reduced CFR, implying that EAT might be involved in the development of CMD [40,41].

A large amount of published data shows a strong association between EAT and AF, which might be explained by the development of an inflammatory atrial myopathy [3,37,38]. Surrounding atrial EAT induces changes in the local atrial environment by persistent inflammation and oxidative stress, modifying ion currents and promoting the development of an atrial arrhythmic substrate with increased AF burden. Additionally, direct fatty infiltration of atrial myocardium by extensions of EAT and atrial fibrosis modulated by bioactive adipokines further contribute to atrial structural remodeling, which in turn maintains arrhythmogenesis [36]. Several studies showed that EAT volume is associated with the prevalence of AF and with its severity, having higher values in persistent or permanent AF compared to paroxysmal AF or sinus rhythm [42,43]. Moreover, periatrial EAT is significantly increased in patients with AF, and it is associated with arrhythmic recurrence after AF ablation, suggesting the direct effect of EAT on the electrophysiologic properties of the adjacent atrial myocardium [44,45].

## 4. The Cause–Effect Relationship between Coronary Microvascular Dysfunction and Atrial Fibrillation

Patients with AF frequently associate anginal symptoms, with demonstrated ischemia and ventricular remodeling in the absence of obstructive CAD [46]. Such manifestations are thought to be the result of altered myocardial perfusion due to CMD induced by AF. This hypothesis was evidenced in the study of Range et al., who evaluated myocardial blood flow (MBF) and coronary vascular resistance (CVR) using PET in patients with AF and nonobstructive CAD compared to controls with sinus rhythm. In the group of patients with AF, MBF at rest, during adenosine-induced hyperemia, was significantly reduced compared to controls. Similarly, hyperemic CVR was significantly increased in patients with AF, outlining the association between CMD and AF. After successful cardioversion with the maintenance of sinus rhythm, normalization of MBF at rest was observed. Although hyperemic MBF and CVR significantly improved following cardioversion, their values remained altered compared to matched controls. These results outline the idea that AF contributes to the development of CMD, which is not completely reversible after restoration of sinus rhythm. Furthermore, the absence of complete CMD resolution after sinus rhythm restoration might suggest that other factors such as an underlying systemic ED might contribute to the progression and maintenance of CMD [7,46].

This assumption is further implied by the work of Wijesurendra et al. who analyzed MBF with CMR at rest and after adenosine in patients with AF and without DM or obstructive CAD compared to matched controls in sinus rhythm, before and after ablation of AF. In the AF group, before ablation, patients had significantly lower baseline and stress MBF relative to controls, suggesting that AF is associated with significant CMD. In addition, the impaired MBF was proportionally correlated with significantly larger LA volumes, and lower LA emptying fraction, advocating for a proportional link between the severity of CMD and ACM. After ablation, baseline and stress MBF did not ameliorate, implying that CMD is not reversible after sinus rhythm restoration. This finding suggests that CMD is not only a direct effect of the AF but may reflect an underlying vascular pathogenic state such as ED. Similarly, the LA emptying fraction remained unchanged after ablation despite considerable reduction in LA maximal volume, reinforcing the existence of an atrial myopathic process with atrial mechanical dysfunction [47].

**Figure 1:**
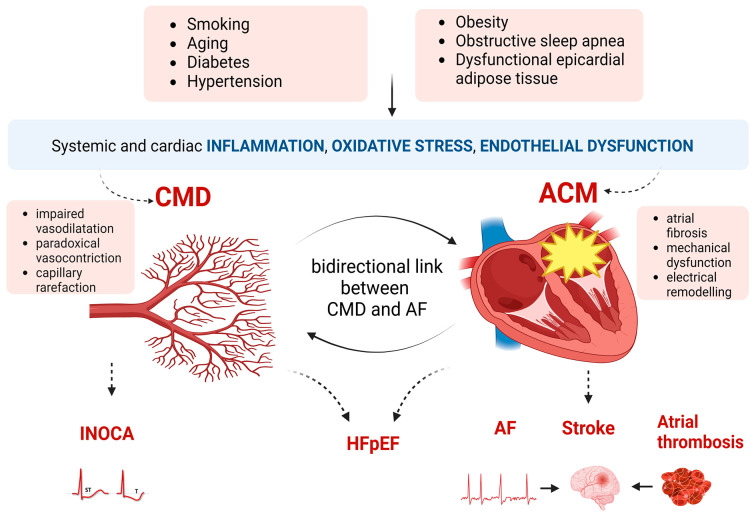
The relationship between CMD, ACM, and ED. ACM, atrial cardiomyopathy; AF, atrial fibrillation; CMD, coronary microvascular dysfunction; ED, endothelial dysfunction; HFpEF, heart failure with preserved ejection fraction; INOCA, ischemia with nonobstructive coronary artery disease. This image was created with BioRender (https://biorender.com/ (accessed on 28 December 2022)).

The relationship between CMD and AF appears, however, to be bidirectional, as illustrated in Figure 1. Alterations in the coronary microcirculation promote atrial fibrosis, creating the substrate for AF. Atrial microvascular dysfunction provides a plausible pathogenic mechanism for ‘lone AF’ in which traditional cardiovascular risk factors causing atrial remodeling are missing. This hypothesis was demonstrated in the study of Skalidis et al. who evaluated the CFR after maximal hyperemia with adenosine in the left circumflex artery and in the left atrial circumflex branch in patients with lone AF and controls in sinus rhythm. They found that in the AF group, CFR in the left atrial circumflex branch was significantly lower than in the left atrial circumflex branch of the controls or in the patients’ left circumflex artery. Their result advanced the idea that isolated atrial microvascular dysfunction is associated with AF, most probably due to atrial ischemia and subsequent structural and electrical atrial remodeling [48]. These observations are further supported by the work of Corban et al. They evaluated coronary endothelial dysfunction (CED): epicardial, defined as a decrease in mid–left anterior descending coronary artery diameter, and microvascular, defined as less than 50% increase in coronary blood flow in response to intracoronary ACh infusions, in patients with angina without obstructive CAD or history of AF. During a follow-up of 10.5 ± 5.5 years, 12% of patients (35 of 300) developed AF, among whom 97% (34 of 35) had been diagnosed with CED at baseline. The presence of CED was associated with an 11% absolute risk increase and a 5.8-fold relative risk increase in AF on follow-up compared to patients with normal baseline coronary endothelial function. These findings suggest that normal coronary endothelial function compared with CED is correlated with a substantially lower absolute and relative risk of long-term incident AF, underlining the possible contribution of CED to the development of AF [6].

## 5. Coronary Microvascular Dysfunction, Atrial Cardiomyopathy, and HFpEF—An Intricate Association

A growing body of evidence lately led to the assumption that cardiac alteration causing HFpEF is a consequence of systemic inflammation, in the context of associated comorbidities (Figure 1) [3,49,50,51,52]. In this regard, it has been demonstrated that circulating inflammatory cytokines and growth factors such as TNFα (tumor necrosis factor α), interleukin-6, and GDF-15 (growth differentiation factor-15) were correlated with HFpEF severity and outcome [53]. Moreover, the recent PROMIS–HFpEF study directly evaluated the ‘comorbidity-inflammation’ paradigm by proteomic analysis. In a cohort of 228 patients with HFpEF, extensive evaluation of inflammatory molecules was performed by quantitation of 47 proteins with already proven association with inflammation and 248 circulating proteins, thereafter disposed in clusters of proteins associated with inflammation. Subsequently, the relationship between inflammation, comorbidity burden, and echocardiographic parameters indicative of HFpEF was attained. The authors found that the comorbidity burden was independently associated with several echocardiographic parameters and multiple biomarkers of inflammation. In addition, systemic inflammation was correlated with echocardiographic parameters of diastolic dysfunction and mediated the association between altered cardiac structure and function and comorbidities, supporting the role of inflammation in the development of HFpEF [51].

Coronary microvascular ED is a central mechanistic pathway leading to HFpEF in the context of chronic inflammation (Figure 1) [52]. Due to high oxidative stress, coronary endothelial synthesis of NO is reduced, which alters paracrine signaling to cardiomyocytes, decreasing the activity of the protein kinase G and ultimately leading to cardiomyocyte hypertrophy and stiffness [52]. Endothelial functional abnormalities are accompanied by coronary microvascular rarefaction, which further promotes myocardial fibrosis and stiffness [54]. The high prevalence of CMD in HFpEF was demonstrated in the PROMISE–HFpEF trial [55]. The authors found that CMD quantitated by CFR < 2.5 with adenosine stress transthoracic Doppler echocardiography was present in 75% of patients with HFpEF and without known unrevascularized CAD. In addition, lower CFR was associated with AF on multivariable analysis and correlated with LA reservoir strain, suggesting the association between CMD and ACM in patients with HFpEF. Moreover, lower CFR was associated with lower RHI measured by PAT, adding proof to the concept of systemic ED possibly leading to CMD and HFpEF. However, the study had limitations since obstructive CAD was not ruled out [55].

Overcoming this limitation, Ozcan et al. directly evaluated the relationship between CMD, AF, and HFpEF in patients undergoing invasive coronary angiography and functional testing with adenosine infusion. CMD was defined as abnormal CFR in the absence of obstructive CAD. The results provided evidence of the clinical association between CMD and AF, as patients with AF were more likely to have abnormal CFR than patients without AF (61% versus 37%). Similarly, patients with CMD tended to have higher rates of AF than patients with normal CFR (32% versus 15%). Interestingly, HFpEF was present in nearly all patients with AF and CMD (91%), and the majority of patients with both AF and HFpEF associated abnormal CFR (71%). What is more, the presence of either CMD, AF, or HFpEF was associated with lower survival free of HF hospitalization at one year [56].

In HFpEF, ACM might result predominantly from high left ventricular filling pressures or might develop synchronously with the LV myopathy secondary to myocardial inflammation and CMD (Figure 1) [57,58]. Interestingly, it appears to exist as a form of ACM disproportionate to the ventricular myopathy and secondary to independent atrial alterations [59]. This concept was advanced for the first time in a secondary analysis of the PROMIS–HFpEF cohort, which aimed to phenotype ACM defined as reduced LA reservoir strain in relation to ventricular myopathy assessed with LV global longitudinal strain (LV GLS) [59]. By calculating the residuals from a linear regression of LA reservoir strain and LV GLS, the authors found that a subgroup of patients had disproportionately greater LA myopathy compared with LV myopathy. This particular phenotype of ACM had distinct echocardiographic features, such as smaller LV and increased biatrial size; it also correlated with less severe diastolic dysfunction but with worse right ventricular systolic function. In addition, disproportionate ACM is associated with worse hemodynamics (lower stroke volume, higher pulmonary artery systolic pressure, and pulmonary vascular resistance) and lower CFR. Finally, disproportionate AM appears to have a specific proteomic profile, as it was associated with five specific proteins related to inflammation, cardiomyocytes, and extracellular matrix alterations, independent of AF [59]. These findings expand the understanding of the potential link between ACM and ventricular myopathy in HFpEF and support the concept of ACM as a distinct pathology in a subgroup of patients with HFpEF.

Concluding the above, there is a clear association between ACM, CMD, and HFpEF, which might reflect a signature profile of cardiac dysfunction in patients with a high comorbidity burden and systemic inflammation [3,56,59].

## 6. New Frontiers in Coronary Microvascular Dysfunction and Atrial Cardiomyopathy—Are Both Part of Systemic Endothelial Dysfunction?

Recently published work led to a new perspective on CMD and ACM, implicating that both are cardiovascular manifestations of a pathogenic systemic state characterized by ED, affecting multiple territories such as the heart, brain, kidney, and peripheral arteries [7,21]. The following sections give an overview of currently available data regarding this complex association.

### 6.1. Coronary Microvascular Dysfunction and Systemic Endothelial Dysfunction

The integration of CMD in the bigger picture of systemic ED is suggested by several studies exploring this potential link. Recently, Al-Badri et al. aimed to assess whether coronary endothelial function might be echoed by peripheral endothelial function in the femoral microcirculation. They included 85 patients, in whom they measured endothelium-dependent coronary epicardial and microvascular function with intracoronary ACh infusions, as well as endothelium-independent pathways with adenosine and sodium nitroprusside. Quantitative coronary angiography was performed before and after each vasoactive infusion, and coronary vascular resistance (CVR) and coronary flow reserve (CFR) were measured. Similarly, femoral vasoactive infusions with ACh and sodium nitroprusside were performed, and femoral vascular resistance (FVR) was measured. The results showed that the percentage change in FVR correlated with the percentage change in CVR in response to ACh, indicating a significant correlation between the magnitude of coronary and femoral microvascular vasodilation induced by ACh. Additionally, there was a significant correlation between changes in the epicardial coronary diameter and femoral microvascular dilation in response to ACh, as patients with epicardial coronary vasoconstriction had attenuated femoral vasodilation compared with those with epicardial coronary vasodilation. Similarly, the changes in CFR with adenosine-reflecting coronary microvascular dilation correlated with the changes in FVR with Ach-reflecting femoral microvascular dilation. Conversely, there was no correlation between the endothelium-independent coronary and femoral microvascular vasodilation in response to sodium nitroprusside infusion [60]. These results advance the idea that peripheral endothelial responses to ACh reflect endothelial function in both the coronary epicardial arteries and microcirculation and the CFR in response to adenosine. Moreover, these findings suggest that evaluation of peripheral endothelial function with ACh at the end of the diagnostic catheterization procedure might indirectly provide insights into the coronary vascular endothelial function and CFR [60].

Another study found that CMD and epicardial vasospasm, assessed by invasive coronary functional testing with ACh and adenosine in patients with INOCA, were associated with systemic microvascular dysfunction, reflected in the altered function of peripheral small arteries from biopsies of subcutaneous gluteal fat, which had reduced maximum relaxation to ACh and increased vasoconstriction in response to ET-1 and a thromboxane agonist [21]. Similar associations between CMD and altered peripheral vascular function measured noninvasively as attenuated RHI or brachial FMD were demonstrated in several other studies [61,62,63].

These results support the concept of ED as a systemic underlying pathogenic process and suggest that the detection of peripheral ED might be a marker of coronary endothelial dysfunction [60].

### 6.2. Atrial Cardiomyopathy and Endothelial Dysfunction

ED is a major contributor to the pathophysiology of the wide spectrum of ACM [4].

First, atrial ED promotes endothelial microthrombi formation with atrial thrombosis even in the absence of atrial arrhythmias, and a wealth of data suggests that ACM might be a potential etiology of embolic strokes of undetermined source (ESUS), which account for up to 30% of ischemic strokes [4,19]. In this regard, several trials showed the absence of a temporal concordance between the detection of atrial arrhythmias and the occurrence of ischemic stroke in patients with implantable cardiac devices [64,65]. In addition, more than half of patients with cryptogenic stroke did not present atrial arrhythmias during three years of continuous heart rhythm monitoring after the cerebral event [66]. Due to the particular distribution on cerebral imaging indicating embolism as a cause of stroke, it has been hypothesized that an underlying prothrombotic atrial substrate might be the cause of the embolic events. Moreover, an interesting study that evaluated thrombus histology from different types of stroke showed that while the composition of cardioembolic and noncardioembolic thrombi had major differences, cryptogenic stroke thrombi had a similar composition to cardioembolic thrombi but not to noncardioembolic ones, implying that ACM might be the source of embolism [67]. This evidence supports the link between ACM and atrial thrombus formation potentially causing ischemic stroke, which might be at least in part mediated by atrial ED [19].

Secondly, atrial ED promotes atrial electrical remodeling and potentiates an arrhythmic atrial substrate, ultimately causing AF. Electron microscopy studies showed that AF is associated with major endothelium ultrastructural changes, such as endothelial cell desquamation and subendothelial edema which further promotes endothelial cell disruption [68]. In addition, endocardial cells in AF suffer an endothelial–mesenchymal transition, leading to increased collagen production, atrial fibrosis, and remodeling, which further maintain the arrhythmia [69].

Multiple studies found an association between AF and circulating biomarkers of ED. VWF, which is produced by endothelial cells in response to endothelial damage and promotes platelet aggregation, has higher levels in patients with AF compared to those in sinus rhythm and correlates with the arrhythmic burden [70,71,72,73]. Moreover, circulating levels of VWF correlate with the degree of endocardial surface damage in electron microscopy [74]. Importantly, VWF has a prognostic role in patients with AF since higher levels of VWF predict MACE and all cause-mortality [75]. Asymmetric dimethyl arginine (ADMA) is another circulating biomarker that acts as a potent inhibitor for endothelial NO synthase (eNOS), leading to decreased NO synthesis and ED. Increased levels of ADMA are associated with AF and predict AF recurrence after cardioversion or ablation [12]. Additionally, in a substudy of the ARISTOTLE trial, ADMA was demonstrated to predict outcomes in anticoagulated patients with AF as plasmatic concentrations increased with higher CHA2DS2-VASc score and were strongly associated with increased mortality [76].

The integration of AF in the systemic state of ED is supported by considerable reports showing correlations with peripheral ED. In line with this, one study demonstrated that patients with permanent AF had lower values of RHI assessed by PAT and a higher burden of inflammatory cytokines compared to controls in sinus rhythm; they also had more comorbidities, substantiating the notion of systemic ED caused by the comorbidity-related inflammation [77]. Several other studies accentuated the direct correlation between AF and impaired endothelial function reflected in lower RHI and brachial FMD [71,78,79,80]. In addition, it appears that the underlying state of ED is associated with the future occurrence of AF since lower values of brachial FMD predict incident AF [81]. On the other hand, AF is associated with lower values of brachial FMD in the absence of comorbidities, suggesting that AF might contribute per se to ED. A plausible mechanism for this causality might be the decreased expression of eNOS leading to lower NO synthesis as a result of altered systemic flow dynamics due to rapid and irregular atrial activity [77,82,83]. Some studies demonstrated an improvement in peripheral vascular function after sinus rhythm restoration with AF cardioversion or ablation [80,84,85,86].

While it is difficult to ascertain which is the primary pathogeny, whether AF or ED, respectively, the abovementioned data underlines the existence of a vicious circle in which ED creates an atrial arrhythmic substrate manifesting as AF, and AF promotes ED at the same time [7].

The main studies describing the interplay between CMD, ACM, and ED are summarized in Table 1.

## 7. Therapeutic Strategies

As evidenced by the above summary, ED is a systemic state which integrates both CMD and ACM development. These pathogenies share common risk factors and comorbidities which cause systemic inflammation and oxidative stress, potentiating ED. Currently, there is little information from randomized controlled trials regarding specific strategies to treat and reverse ED. Considering the shared systemic risk factors which promote ED, it appears legitimate that preventive lifestyle interventions such as smoking cessation and weight loss, and controlling comorbidities such as arterial hypertension, DM, and dyslipidemia, could ameliorate ED and the cardiovascular damage associated with it.

**Figure 2:**
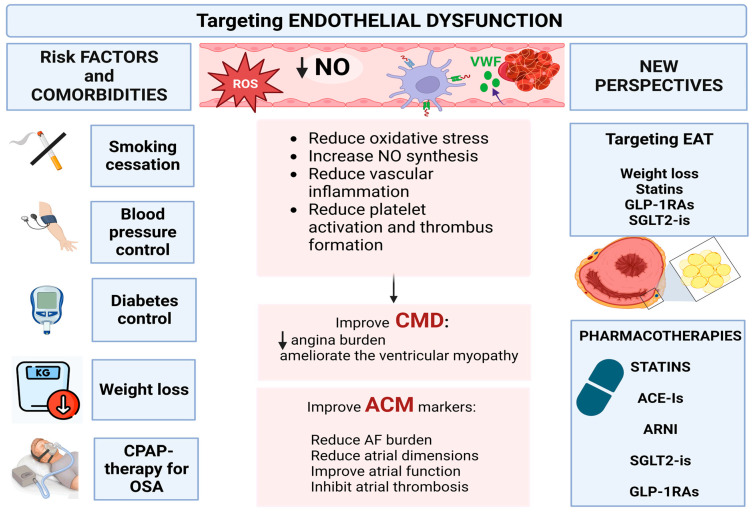
Targeting endothelial dysfunction: a strategy to improve CMD and ACM. ACM, atrial cardiomyopathy; AF, atrial fibrillation; ACE-Is, angiotensin-converting enzyme inhibitors; ARNI, angiotensin receptor–neprilysin inhibitor; CMD, coronary microvascular dysfunction; CPAP, continuous positive airway pressure; EAT, epicardial adipose tissue; GLP-1RAs, glucagon-like peptide 1 receptor agonists; NO, nitric oxide; OSA, obstructive sleep apnea; ROS, reactive oxygen species; SGLT2-is, sodium–glucose cotransporter 2 inhibitors; VWF, von Willebrand Factor. This image was created with BioRender (https://biorender.com/ (accessed on 27 January 2022)).

In the following section, we discuss the impact of weight loss and CPAP therapy for OSA on systemic ED, CMD, and ACM manifestations. We also provide current knowledge on potential actions that target EAT. Finally, we analyze the effect on ED of pharmacotherapies used to treat arterial hypertension, diabetes, and dyslipidemia, and assess whether they might have a role in ameliorating CMD and ACM markers. Figure 2 provides a model for a therapeutic strategy targeting ED and potentially improving CMD and ACM manifestations.

### 7.1. Targeting Obesity

Obesity is a major contributor to the development of vascular ED, and multiple studies have demonstrated that weight loss could significantly improve it [87,88,89].

A recent meta-analysis showed that obese patients who had undergone bariatric surgery had significant improvement in endothelial function ascertained by increasing FMD measurements, independent of the weight loss, suggesting that weight-independent mechanisms such as endocrine and incretin-mediated effects and improved inflammatory status might contribute as well to the amelioration of ED [90].

The beneficial effect of weight loss translates into improved cardiovascular outcomes with a decrease in the incidence of AF. In a trial, patients with a weight loss > 10% had a 6-fold higher probability of arrhythmia-free survival compared with those with <10% weight loss [91]. At the same time, increased weight loss was associated with the reversal of AF type and progression since 88% of the patients with >10% weight loss had AF reversal from persistent to paroxysmal or no AF [92]. Another trial underlined the positive impact of improved cardiorespiratory fitness on AF, showing that a cardiorespiratory fitness gain of >2 METs in obese patients with AF undergoing a tailored exercise program led to a significant decrease in AF burden and symptom severity compared to those with <2 METs gain in exercise capacity [93].

Similarly, it appears that weight loss and improved exercise capacity ameliorate CMD, reflected in increases in CFR assessed by Doppler echocardiography of the left anterior descendent artery [94,95]. Conversely, another trial showed that major weight loss resulted in significantly improved angina symptoms among women with microvascular angina but without amelioration of coronary microvascular function [96]. Further studies are needed to systematically determine the impact of weight loss on CMD.

### 7.2. Targeting Obstructive Sleep Apnea: The Role of CPAP Therapy

ED is an early anomaly in OSA, and continuous positive airway pressure (CPAP) therapy could potentially modulate it.

A meta-analysis evaluating this effect found a significant improvement in peripheral ED with CPAP use, noted in an important increment in brachial FMD of 3.9%, whereas no significant change was observed in the group with standard therapy or sham CPAP [97]. Similar augmentation of FMD after 3 months of CPAP therapy in patients with OSA was observed in another study, which also showed a decrease in circulating biomarkers of endothelial damage and an increase in vascular endothelial growth factor as a marker of vascular repair [98].

There is little evidence regarding the impact of CPAP therapy in patients with OSA and CMD. A study showed that short-term (2 weeks) discontinuation of therapeutic CPAP in moderate to severe OSA did not affect the coronary microvascular function, assessed with adenosine-induced MBF using PET [99]. Another small-size study showed a significant and acute increase in CFR assessed by transthoracic Doppler echocardiography with adenosine-induced hyperemia after just one night of CPAP therapy [100]. Given the potential of CPAP therapy to reverse ED, further studies evaluating the impact of CPAP therapy on coronary microvasculature dysfunction would be of major significance.

CPAP therapy appears to exert a benefic effect on ACM substrate. This concept is illustrated by the results of the SLEEP–AF study in which patients with AF and at least moderate OSA had improved parameters at electrophysiologic study after CPAP therapy compared to baseline electrophysiologic evaluation [101]. Similarly, another study showed that CPAP therapy is associated with improved LA function after 24 weeks of administration compared to baseline and sham CPAP [102]. In the same line, several meta-analyses showed that patients with OSA who receive CPAP therapy have lower rates of arrhythmia recurrence after AF catheter ablation or cardioversion, while other studies did not find a benefit for those treated with CPAP versus usual care [103,104,105,106]. Conversely, the SAVE trial which tested the efficiency of CPAP therapy in the secondary prevention of cardiovascular events in patients with OSA and established vascular diseases did not show an improvement in outcomes or a reduction of new-onset AF risk in the CPAP group compared to controls [107]. While CPAP therapy contributes to a reduction of the atrial arrhythmic substrate and LA positive remodeling, it remains to be established in further trials the extent of this effect and how it can be augmented.

### 7.3. Focusing on Epicardial Adipose Tissue—A New Therapeutic Target?

As previously stated, EAT creates an inflammatory environment that promotes CMD and AF. In line with this, reducing epicardial fat emerges as a potential therapeutic target to counteract coronary and atrial inflammation, and to decrease the risk of subsequent morbidity [3].

Several studies demonstrated that significant weight loss following bariatric surgery is associated with a reduction in EAT [108,109]. Moreover, this effect is maintained over time, as shown in a study in which patients with bariatric surgery had 30% lower epicardial fat compared to severely obese patients without the surgical procedure after a follow-up of 11 years [110].

Various pharmacotherapies with direct or indirect action on adiposity might reduce or improve the function of EAT.

Statin therapy appears to act on epicardial fat causing its reduction, independent of the cholesterol-lowering effect, which might suggest that other mechanisms such as their anti-inflammatory properties might be implicated [111,112]. Consistent with this hypothesis, it has been shown that EAT attenuation on computed tomography, which is a marker for the metabolic activity of adipose tissue, decreased significantly after 1 year of statin administration, irrespective of the decrease in the lipid fractions, implying that statins lower the metabolic activity of the epicardial fat [113].

Glucagon-like peptide 1 receptor agonists (GLP- 1RAs) are a class of drugs originally designed for the treatment of type 2 diabetes but are now also used to treat obesity due to their weight loss benefits. This additional effect is achieved secondary to GLP- 1RAs property of reducing the appetite and the sensation of hunger, as well as increasing satiety [114]. Liraglutide is a representative of the class which has been approved for the treatment of obesity, following the results of the SCALE trial [115]. Additionally, liraglutide demonstrated cardioprotective effects, with a reduction in MACE and all-cause mortality in the LEADER trial [116]. However, the mechanisms underlying the cardiovascular benefit of liraglutide are not completely elucidated. One possible mechanistic explanation could be the ability of GLP- 1RAs to modulate EAT and therefore decrease surrounding heart inflammation. In this regard, Iacobellis et al. showed that in diabetic patients liraglutide reduced the EAT by 29% and 36% at 3 and 6 months, respectively, while sole metformin administration did not modify EAT [117]. This additional effect of liraglutide raises the question of whether this medication could be used to specifically reduce epicardial fat and thus decrease myocardial and coronary inflammation mediated by adiposity.

Sodium–glucose cotransporter 2 inhibitors (SGLT2-is) are a revolutionary class of drugs due to their strong cardio–renal protective effect. While initially addressed to diabetic patients for their glucose-lowering action, they are currently one of the main pillars in the treatment of heart failure, as they demonstrated in large clinical trials a significant reduction in cardiovascular death and heart failure hospitalization [118]. SGLT2-is act by preventing glucose and sodium reabsorption in the nephron, therefore inducing osmotic diuresis [118]. Additionally, they cause moderate weight loss and visceral fat reduction, most probably secondary to caloric loss induced by glycosuria [119]. More recently, their effect on EAT has been investigated. In a study, diabetic patients with a body mass index > 27 kg/m^2^ were randomly assigned to receive dapagliflozin in addition to metformin, or metformin alone. The results showed a significant decrease of EAT in the dapagliflozin group by up to 20% from baseline to 24 weeks, whereas in the metformin group the reduction in EAT was smaller. Interestingly, EAT thickness reduction assessed echocardiographically was significantly greater than body weight loss (20% vs. 8% at week 24, respectively) which might suggest the contribution of alternative pathways independent of weight loss to epicardial fat reduction [120]. Several studies demonstrated similar results with other SGLT2-is reducing EAT, underlying a potential drug class effect [121,122]. Conversely, in the EMPACEF study, there was no change in the epicardial fat volume assessed by MRI after 12 weeks in diabetic patients receiving empagliflozin 10 mg daily compared to placebo treatment [37]. However, this result does not exclude the benefic effect of SGLT2-is on EAT at a molecular level. Diaz Rodriguez et al. demonstrated that the action of SGLT-is on EAT is beyond macroscopic fat changes. They showed that SGLT2 is expressed in EAT and that dapagliflozin increased glucose uptake and glucose transporter type 4 at this level. Moreover, dapagliflozin reduced the secretion of pro-inflammatory chemokines and promoted the healing of human coronary artery endothelial cells [123]. Another study demonstrated that dapagliflozin reduced the anaerobic glucose metabolism, and consequently the release of lactate and acidosis in epicardial fat, without increasing lipogenesis [124].

### 7.4. Pharmacotherapies with a Potential Role on Peripheral, Coronary, and Atrial Endothelial Dysfunction

#### 7.4.1. Statins

In addition to their lipid-lowering effect, statins have multiple pleiotropic properties that not only slow the atherosclerosis progression but also induce positive changes in the coronary microvasculature. Such mechanisms include reducing inflammation and oxidative stress, silencing profibrotic pathways, and improving endothelial function, which contribute altogether to vascular endothelial health [125].

The effect of statins on ED was suggested by early studies which showed increases in brachial FMD after statin treatment [125]. A potential contribution of statins to coronary vasodilatation was suggested by several studies showing an improvement in CFR after treatment with statins, assessed by PET or Doppler echocardiography [126,127]. Another study performing invasive coronary physiology evaluation after high-intensity statin therapy in patients with moderate CAD demonstrated modest improvements in coronary microvascular function with an increase in CFR and a decrease in hyperemic microvascular resistance (HMR), advocating for a role of statins in improving CMD beyond coronary atheroma regression [128]. A meta-analysis addressing the effect of statins on peripheral and coronary endothelial function assessed non-invasively and invasively demonstrated significant improvement in both peripheral and coronary endothelial function [129]. However, none of the above studies evaluated the effect of statins on coronary physiology in the specific group of INOCA patients. Nevertheless, data coming from registries showed that this population has higher than expected rates of MACE, which underlines the importance of adequate diagnosis and treatment [130]. Integrating the known benefit of statins on all-cause mortality among patients with CAD and the associated positive effect on endothelial function, statins are part of the therapeutic strategy in patients with INOCA and CMD. The ongoing trial WARRIOR will evaluate whether intensive medical therapy with high-intensity statins and angiotensin-converting enzyme inhibitors (ACE-Is) or angiotensin receptor blockers (ARBs) will significantly reduce MACE in this population, providing evidence for the use of statins in INOCA patients [131].

Experimental and observational studies suggested a potential antiarrhythmic effect of statins, demonstrating that these drugs protect against AF [132]. Possible pathways to explain these observations involve antioxidative actions, inflammation attenuation, and epicardial fat reduction induced by statins [132,133]. Indeed, several large meta-analyses evidenced that statins were significantly associated with a decreased risk of AF compared with control. In addition, the benefit of statin therapy appeared to be higher in secondary prevention than for new-onset or postoperative AF [132,134,135]. Apart from preventing atrial electrical remodeling, statins appear to modulate extracellular atrial components, counteracting distortion of atrial structure [136]. Moreover, statins might improve left atrial mechanical function, as suggested by a study demonstrating significant improvement in echocardiographic parameters of LA function after statin therapy [137].

Interestingly, the potential benefit of statins on both CMD and ACM, which are pathologies frequently associated with HFpEF, as part of the systemic ED, might explain the beneficial effect of statins on mortality observed in HFpEF patients without CAD, which is not translated in the population of patients with heart failure with reduced ejection fraction (HFrEF) [138,139].

#### 7.4.2. Renin–Angiotensin–Aldosterone System Blockade

ED is strongly linked to RAAS activation, which causes vasoconstriction, endothelial cell proliferation, and vascular oxidative stress. Indeed, ample evidence suggests that RAAS blockers can improve ED. A meta-analysis showed that treatment with ACE-Is significantly improved brachial FMD versus placebo or no treatment. Additionally, ACE-Is were more efficacious in improving brachial FMD when compared with other antihypertensive therapies, and there was no significant difference between ACE-I and ARB effect on brachial FMD [140]. Similar results illustrating improvements in ED with ACEI-Is and ARBs were presented by other studies [141,142].

ACE-Is are demonstrated to impact coronary microvascular function, most probably by various mechanistic pathways, such as improved systemic hemodynamics and coronary endothelial function, and blockade of the local deleterious effects of angiotensin II. The potential role of ACE-Is in the management of CMD was illustrated in the WISE study, which included women with INOCA and CMD defined by a CFR < 3, randomized to receive quinapril or placebo for 16 weeks. CFR was assessed at baseline and after completion of treatment with invasive intracoronary Doppler measurements, before and after adenosine infusion. The results showed that CFR improvement was higher in the ACE-Is group and that the benefit was greater in patients with lower baseline CFR (≤2.5). In addition, women receiving ACE-Is had significantly improved anginal symptoms compared to placebo [143]. The same benefit on CMD in patients with INOCA was present in a study with another ACE-I, enalapril, which was associated with increased CFR values and decreased circulating biomarkers of ED [141]. Other studies added evidence to the positive effect of ACE-Is on CMD associated with comorbidities such as arterial hypertension or DM [144]. Further data regarding the role of ACE-Is in the long-term outcome of INOCA patients will be obtained after the completion of the WARRIOR trial [131].

RAAS activation has a major contribution to the development of ACM by activating proinflammatory and profibrotic pathways [7,145]. Experimental evidence suggested that RAAS blockade could modify the atrial substrate and prevent AF by opposing the adverse effects of angiotensin II [146]. Consequently, it has been hypothesized that RAAS-blocking therapies could play a role in the primary prevention of AF. Indeed, large randomized controlled trials evaluating RAAS inhibition in congestive HF (TRACE, Val-HeFT, CHARM) demonstrated that treatment with an ACE-I or ARB significantly reduced the risk of new-onset AF [146]. Hypertensive patients treated with RAAS inhibitors also associate a smaller risk of new-onset AF and present improvements in peripheral endothelial function [140,146]. Conversely, the potential benefic role of RAAS inhibition in the secondary prevention of AF was not consistent among studies [147]. RAAS blockade appears to improve atrial structural remodeling, with a lower expression of collagen and capillary rarefaction on left atrial biopsies from AF patients treated with ACE-Is compared to AF patients without ACE-I therapy [148]. In addition, RAAS blockers might induce a reduction in LA dimensions as suggested in an analysis from the LIFE study, which showed that LA diameter was more markedly reduced by losartan compared to atenolol and that the reduction in LA diameter was associated with a smaller risk of new-onset AF [149].

#### 7.4.3. Angiotensin Receptor–Neprilysin Inhibitor

Sacubitril/valsartan is the first drug in the class of the angiotensin receptor–neprilysin inhibitors (ARNI). Its mechanisms of action include both blocking RAAS activation with valsartan, an ARB, and preserving the natriuretic peptides (NPs) by inhibiting their degradation with sacubitril, a neprilysin inhibitor. The NP system includes hormones such as atrial NP (ANP) and B-type NP (BNP), which are secreted by cardiomyocytes in response to myocardial wall stretch, as well as C-type NP (CNP), which is mainly produced by endothelial cells [150]. ANP and BNP promote natriuresis, diuresis, and systemic vasodilatation, which all contribute to the reduction in myocardial wall stress. Moreover, they have beneficial local cardiac autocrine and paracrine effects as they inhibit the proliferation of cardiac fibroblasts and exert anti-inflammatory effects on monocytes, preventing both ventricular and atrial fibrosis [150].

Recent data suggest that endothelial CNP contributes to the modulation of distal arteriolar and capillary blood flow by mediating the cross talk between endothelial cells and pericytes [151]. In addition, it enhances the local effect of NO on vascular tone, promoting endothelial-dependent vasodilatation [152]. Therefore, preventing CNP degradation with sacubitril/valsartan could improve endothelial function and thereby augment endothelial-dependent vasodilatation. In this regard, a recent study that included 100 patients with HFrEF showed for the first time that ARNI improves ED and the proinflammatory and prothrombotic states associated with it. The authors showed that a 6-month treatment with sacubitril/valsartan resulted in the betterment of impaired vasodilatation, with a significant RHI increase. Additionally, ARNI therapy was associated with a decrease in serum levels of oxidative stress and platelet activation biomarkers, as well as inflammatory cytokines, all of these mechanisms accounting for an improvement in vascular function [153].

While there are no data regarding sacubitril/valsartan effects on CMD, we could assume that it may potentially improve it given its mechanisms of action, which include blocking the deleterious vascular effects of angiotensin II with valsartan and preserving the vascular and endothelial benefits promoted by NPs with sacubitril. A study evaluating this hypothesis could provide interesting results.

Apart from promoting positive ventricular remodeling which translates into improved outcomes in patients with HF, emerging evidence suggests that ARNI could be beneficial in ACM as well. Through their antihypertrophic and antifibrotic effects, NPs prevent atrial structural remodeling and mechanical dysfunction. By counteracting atrial fibrosis, NPs contribute to normal electrical impulse generation and conduction, indirectly exerting an antiarrhythmic effect [150]. Moreover, ANP is directly involved in atrial arrhythmogenesis, as ANP gene variants are associated with AF, including a familial form of this arrhythmia [154,155]. An experimental study using a rabbit model with AF demonstrated that sacubitril/valsartan attenuates atrial electrical remodeling, as shown by reduced AF inducibility and restoration of the atrial effective refractory period, most probably due to the reduction of calcium overload and molecular signaling associated with electrical atrial instability. In addition, the administration of sacubitril/valsartan attenuated atrial structural remodeling by preventing atrial enlargement and reducing atrial myocardial fibrosis [156]. Similarly, another animal study showed that in rats with subcutaneous stimulation with angiotensin II, administration of sacubitril/valsartan attenuated atrial fibrosis and reduced susceptibility to AF [157]. Data coming from human studies demonstrate positive effects of ARNI in ACM as well. A study showed that patients who received sacubitril/valsartan after radiofrequency catheter ablation of AF displayed a significant decrease in the left and right atrial diameter and volume index from baseline to 24 weeks after ablation, while this effect was not present in patients who received only valsartan [158]. In the same line, another recent study demonstrated that patients that were receiving sacubitril/valsartan were significantly more likely to have successful electrical cardioversion for persistent AF and subsequent maintenance of sinus rhythm compared to patients without ARNI treatment [159]. These results highlight additional clinical benefits for ARNI, namely their potential role to reverse or attenuate the atrial cardiomyopathic process and to prevent AF recurrence. However, whether sacubitril/valsartan is effective in the upstream therapy of AF is not entirely elucidated. A recent meta-analysis of six randomized control trials evaluated the effect of sacubitril/valsartan in the prevention of AF occurrence in patients with HF and demonstrated that sacubitril/valsartan is similar to either enalapril or valsartan in the prevention of AF occurrence in patients with HF [160]. Notwithstanding, none of these trials were designed to primarily assess the effect of sacubitril/valsartan on the occurrence of new-onset AF, and further trials are needed to evaluate this concept.

#### 7.4.4. Sodium–Glucose Cotransporter 2 Inhibitors

Beyond their metabolic benefits, SGLT2-is determine a myriad of pleiotropic effects such as an increase in hematocrit, lower values of uric acid and urine albumin: creatinine ratio, improvement in the tubuloglomerular function, and a reduction of inflammation and oxidative stress [161,162,163]. Emerging evidence suggests that SGLT2-is may play a role in the modulation of ED, which might contribute to their cardio–renal protection.

Several studies tested the effectiveness of SGLT2-is on peripheral ED. In the DEFENCE study, dapagliflozin on top of metformin led to a significant improvement of FMD in type 2 DM patients compared to single therapy with metformin. Moreover, dapagliflozin reduced oxidative stress, as suggested by lower urinary levels of oxidative stress biomarkers [164]. Data from Solini et al. showed that dapagliflozin acutely improved ED as suggested by an increase in brachial FMD after a 2-day treatment with dapagliflozin 10 mg, while this effect was not found after a 4-week treatment [165,166]. Interestingly, in another study, serum endothelial microparticles significantly increased following a 12-week treatment with empagliflozin in overweight/obese women with polycystic ovary syndrome, suggesting increased endothelial activation, while RHI did not change from the baseline assessment. These discordant findings might be explained by an initial transitory, regenerative endothelium response to a potentially positive therapeutic intervention [167].

There is little data specifically describing the effect of SGLT2-is on coronary microvasculature. A recent study analyzed the effects of empagliflozin by evaluating heart biopsies from HFpEF patients as well as from rats that were treated in vivo [168]. Increased microvascular inflammation and markers of oxidative stress were present on both human and murine heart specimens, which were significantly attenuated with empagliflozin. In addition, empagliflozin significantly improved endothelial vasorelaxation induced by ACh without affecting smooth muscle vasorelaxation, suggesting that empagliflozin might ameliorate the microvascular endothelial dysfunction from HFpEF. Moreover, empagliflozin reversed cascade molecular pathways induced by inflammation and oxidative stress which contribute to cardiomyocyte stiffness and diastolic dysfunction in HFpEF [168]. Interestingly, another study demonstrated that dapagliflozin provides microvascular protection by reducing ED and microvascular damage mediated by ischemia–reperfusion cardiac injury, therefore being a potential therapeutic strategy to limit myocardial infarct formation and expansion [169]. As experimental studies led to the hypothesis that SGLT2-is might improve myocardial perfusion, this concept was directly tested in the SIMPLE trial, which evaluated the impact of empagliflozin treatment versus placebo on myocardial flow reserve (MFR), directly quantitated by positron emission tomography/computed tomography (PET/CT) at rest and during adenosine stress, in patients with type 2 DM and ischemic heart disease or other cardiovascular risk factors. The results showed no change in MFR after empagliflozin treatment and did not find any treatment effect [170]. However, these outcomes cannot be generalized to the whole spectrum of CMD as this study did not evaluate endothelial-dependent pathways, nor CMD without CAD, which in fact might represent the population with the greatest benefit from SGLT2-is. A study evaluating this hypothesis could provide interesting results.

Extending the effects of SGLT2-is, it appears that these drugs could potentially target the pathogenic atrial substrate from ACM as illustrated by several experimental studies. In line with this, a study using a diabetic rat model demonstrated that empagliflozin has incremental positive effects on left atrium structure and function. Specifically, empagliflozin administration in diabetic rats was associated with significantly smaller LA diameters and with attenuated LA interstitial fibrosis and atrial myocyte hypertrophy; moreover, it prevented DM-induced atrial electrical remodeling and AF inducibility, and it improved the mitochondrial function [171]. In another animal study, canagliflozin prevented atrial remodeling by suppressing AF inducibility and reducing atrial interstitial fibrosis and oxidative stress [172]. The atrial positive remodeling with SGLT2-is could be the result of the pleiotropic effects of SGLT2-is, such as improved atrial endothelial function, reduction of low-grade inflammation, and oxidative stress. In a subanalysis from the DECLARE-TIMI 58 trial, dapagliflozin reduced the risk of a first AF/atrial flutter (AFL) event during follow-up by 19% and the number of total AF/AFL events by 23%. This effect was consistent regardless of the patient’s previous history of AF, atherosclerotic cardiovascular disease, or HF [162]. In addition, several studies showed that SGLT2-is reduce the risk of new-onset AF and the incidence of intracardiac thrombosis, which sets a new direction for the use of SGLT2-is in ACM [173,174,175]. Conversely, in an analysis from the DAPA-HF trial, dapagliflozin did not reduce the risk of new-onset AF [176]. Alongside reverse ventricular remodeling with a reduction in left ventricle volumes and mass, SGLT2-is demonstrated to induce positive atrial remodeling with a reduction in LA dimensions [177]. Considering the undeniable beneficial effects of SGLT-is in HF studies, it seems legitimate to assume that these are at least in part due to improvements in LA function.

#### 7.4.5. Glucagon-like Peptide 1 Receptor Agonists

GLP-1RAs demonstrated conflicting results regarding the potential benefit on systemic ED. While improving endothelial function markers in experimental research, liraglutide did not significantly increase endothelium-dependent vasodilation assessed by measuring forearm blood flow using venous occlusion plethysmography after 12 weeks in a cohort of diabetic patients without other cardiovascular diseases [178]. Similar results came from another GLP1-RA, exenatide, which displayed no differences in the change in RHI when compared to metformin in obese patients with pre-diabetes [179]. Conversely, Lambadiari et al. showed that diabetic patients treated with liraglutide for 6 months had increases in FMD compared to baseline and had a greater percentage difference in FMD compared to the group treated with metformin [180]. The same group showed that a twelve-month treatment with liraglutide, empagliflozin, or both increased endothelial glycocalyx thickness assessed noninvasively by measuring the perfused boundary region of the sublingual arterial microvessels using a dedicated camera, suggesting potential improvement in endothelial function [181]. In addition, the combination therapy had a 3-fold higher increase of the endothelial glycocalyx and a greater improvement in myocardial performance echocardiographic indices. These results advance the idea that a combination therapy between a SGLT2-i and a GLP-1RA might provide greater cardiac and vascular protection than single therapy alone [181].

GLP-1RAs are demonstrated to reduce the rates of cardiovascular mortality and myocardial infarction, and improvements in CMD might be a potential mechanism [116]. In an experimental study, liraglutide reduced the infarct size and improved cardiac output after induction of myocardial infarction in liraglutide-treated mice [182]. In patients with STEMI undergoing PCI, administration of exenatide at the time of reperfusion was protective against ischemia–reperfusion injury [183]. However, the identification of glucagon-like peptide 1 receptor (GLP-1R) expression in coronary artery endothelial cells and smooth muscle cells varied across studies, and the conditions that induce GLP-1R expression at this level need to be further elucidated [184]. A study evaluating the short-term impact of liraglutide on coronary microcirculation showed a small increase in CFR assessed by transthoracic Doppler echocardiography during dipyridamole induced stress but with no difference in effect when compared to the group with no treatment [185]. Similarly, another study showed that liraglutide did not improve symptoms and CFR assessed with adenosine infusion in obese non-diabetic women with CMD and INOCA [186]. It is worth mentioning that these studies had limitations, such as the small size of the study population and patients not being on liraglutide on final CFR assessment, respectively, which might have impacted the results. Further studies including a larger number of patients followed for a longer period of time and with more severe CMD are needed to assess the effect of GLP1-RAs on CMD. Interestingly, a subanalysis from the LIRAFLAME trial proved for the first time that liraglutide reduces coronary artery inflammation quantitated with multimodality PET imaging in patients with atherosclerotic CAD, which could suggest a potential benefit of GLP1-RAs on coronary microvascular inflammation as well [187]. Further research is needed to establish to what extent GLP-1RAs can potentially improve CMD.

Since GLP-1RAs demonstrated to reduce EAT volume and its metabolic activity, we could assume that they ameliorate periatrial inflammation with a positive effect on atrial arrhythmias [117]. An experimental study showed that liraglutide suppresses AF inducibility [188]. In a recent meta-analysis of the two randomized controlled trials of semaglutide, PIONEER and SUSTAIN, semaglutide demonstrated to decrease the risk of AF by 31% [189]. Conversely, another study did not show a reduction in the risk of new-onset AF with GLP-1RAs [174]. Concern was initially raised upon a potential increased risk of AF associated with GLP-1RAs, but this observation was dismissed by subsequent research [190].

## 8. Clinical Significance and Future Perspectives

Of particular importance for the clinician is the clinical recognition of the profile of the patient with multiple comorbidities, INOCA, and/or manifestations of ACM as it could provide a more accurate and comprehensive estimation of the patient’s prognosis and risk of future cardiovascular events. Both CMD and ACM, most frequently manifesting as AF, may be part of the same underlying disease process represented by endothelial dysfunction and may potentiate each other. Future studies are needed to further evaluate these associations and to systematically investigate the causality relationship between CMD and AF. Controlling cardiovascular risk factors which cause endothelial dysfunction improves these conditions, and their particular contribution to the long-term prognostic benefit remains to be established in future studies. Endothelial dysfunction emerges as a new therapeutic target, and further research is needed to assess to what extent reversing this common pathophysiological mechanism could indeed improve CMD and ACM manifestations. Certain pharmacotherapies such as statins and ACE-Is have been shown to positively influence CMD and ACM. ARNI, SGLT2-is, and GLP-1RAs are newer therapies which may have the potential to improve coronary and atrial endothelial dysfunction as well. Further studies are needed to clarify whether these drugs act on a common pathogenic substrate and whether their administration translates into long-term positive outcomes. This concept merits indeed further exploration as it could provide a new understanding of the treatment of these pathogenies, centered on targeting their common underlying pathogenic process. In the same line, of major significance would be to evaluate whether administration of these therapies in patients with a high comorbidity burden and endothelial dysfunction but without clinically manifesting CMD or ACM could potentially prevent their development, thus decreasing related morbidity and improving patient’s prognosis.

## 9. Conclusions

CMD causing INOCA and ACM share similar risk factors and are frequent encounters in patients with a high comorbidity burden. CMD and AF are often found in association, and one may promote the other. Both pathologies can lead to HFpEF, and endothelial dysfunction is a common mechanistic pathway. Targeting endothelial dysfunction emerges as a new therapeutic strategy that could improve cardiovascular morbidity. Nonpharmacological measures such as weight loss and CPAP therapy for OSA ameliorate systemic endothelial dysfunction and can reduce atrial and vascular adverse remodeling. Targeting epicardial adipose tissue could reduce periatrial and coronary microvasculature inflammation and dysfunction. Certain therapies such as statins, ACE-Is, ARNI, SGLT2-is, and GLP-1RAs demonstrated a potential beneficial effect on endothelial dysfunction, reversing this pathogenic substrate and improving CMD and ACM manifestations.

## Figures and Tables

**Table 1:** Main studies and clinical trials describing the CMD, ACM, and ED association.

Concept	Study/Clinical Trial	Evaluated Parameters	Main Findings	Conclusions/Clinical Implications
CMD and AF-a bidirectional link	Range et al. [46]	→ MBF and CVR using PET at rest and after adenosine, in patients with AF and nonobstructive CAD, compared to controls in SR	→ patients with AF had significantly reduced MBF and significantly increased CVR → hyperemic MBF and CVR significantly improved following cardioversion, but their values remained altered compared to controls in SR	→ AF promotes CMD, which is not completely reversible after restoration of SR
	Wijesurendra et al. [47]	→ MBF with CMR at rest and after adenosine, in patients with AF and with nonobstructive CAD, compared to controls in SR, before and after ablation of AF	→ patients with AF had significantly lower baseline and stress MBF relative to controls, which did not ameliorate after AF ablation → impaired MBF was proportionally correlated with significantly larger LA volumes, and lower LA emptying fraction	→ AF is associated with significant CMD → CMD is not reversible after SR restoration → CMD and ACM severity are proportionally associated
	Skalidis et al. [48]	→ CFR after adenosine in the left circumflex artery and in the left atrial circumflex branch in patients with lone AF and controls in SR	→ in the AF group, CFR in the left atrial circumflex branch was significantly lower than in the left atrial circumflex branch of controls in SR, or in the patients’ left circumflex artery	→ isolated atrial microvascular dysfunction is associated with AF
	Corban et al. [6]	→ invasive evaluation of coronary endothelial dysfunction (CED), epicardial and microvascular, with intracoronary ACh infusions, in patients with angina, without obstructive CAD or history of AF	→ during a follow-up of 10.5±5.5 years, 12% of patients developed AF, among whom 97% had been diagnosed with CED at baseline → CED was associated with an 11% absolute risk increase and a 5.8-fold relative risk increase in AF on follow-up	→ normal coronary endothelial function compared with CED is correlated with a substantially lower absolute and relative risk of long-term incident AF
The association between CMD, ACM, and HFpEF	Sanders-van Wijk et al. [51] (PROMIS–HFpEF trial)	→ inflammatory molecules and echocardiographic parameters indicative of HFpEF, in a cohort of 228 patients with HFpEF	→ Systemic inflammation linked to a high comorbidity burden is associated with HFpEF	
	Shah et al. [55] (PROMIS–HFpEF trial)	→ CFR with adenosine stress transthoracic Doppler echocardiography in patients with HFpEF	→ CFR <2,5 was present in 75% of patients with HFpEF → lower CFR was associated with AF and correlated with LA reservoir strain	→ CMD is highly prevalent in HFpEF → CMD and ACM co-exist in patients with HFpEF
	Ozcan et al. [56]	→ CFR with adenosine, in patients undergoing invasive coronary angiography in the absence of obstructive CAD → directly evaluated the relationship between CMD, AF, and HFpEF	→ patients with AF were more likely to have abnormal CFR than patients without AF (61% versus 37%) → patients with CMD tended to have higher rates of AF than patients with normal CFR (32% versus 15%) → HFpEF was present in nearly all patients with AF and CMD (91%) → the majority of patients with both AF and HFpEF associated abnormal CFR (71%)	→CMD, AF, and HFpEF are often found in association, suggesting common mechanistic pathways and reciprocal interactions
	Patel et al. [59] (PROMIS–HFpEF trial)	→ LA reservoir strain (surrogate for LA cardiomyopathy) → LV global longitudinal strain (surrogate for LV myopathy) in patients with HFpEF	→ a subgroup of patients had disproportionately greater LA myopathy compared with LV myopathy → disproportionate ACM is associated with worse hemodynamics and lower CFR	→ disproportionate ACM might represent a distinct pathology in a subgroup of patients with HFpEF
CMD and ED	Al-Badri et al. [60]	→ CVR and CFR with ACh and adenosine → FVR with ACh	→ peripheral endothelial responses to ACh reflect endothelial function in both the coronary epicardial arteries and microcirculation and the CFR in response to adenosine	→ peripheral endothelial function with ACh could be used to indirectly assess the coronary endothelial function and CFR with adenosine
	Ford et al. [21]	→ CMD and epicardial vasospasm, with ACh and adenosine in patients with INOCA → peripheral small arteries (from biopsies) vasodilator response to ACh and vasoconstrictor response to ET-1 and a thromboxane agonist	→ CMD and epicardial vasospasm were associated with peripheral ED	→ INOCA (CMD and epicardial vasospasm) might be part of a systemic disease characterized by ED
	Nardone et al. [61]	→ CMD with ACh and adenosine → RHI and brachial FMD	→ RHI and FMD were attenuated in patients with CMD	→ peripheral ED is associated with CMD → altered FMD and RHI could be useful to screen for CMD in patients with chest pain and normal coronary angiograms
	di Serafino et al. [62]	→ IMR with adenosine → RHI	→ RHI was lower in patients with coronary microvascular impairment (IMR > 25)	
	Park et al. [63]	→ CFR in patients with nonobstructive CAD → brachial FMD	→ FMD was decreased in patients with abnormal CFR	
ACM and ED	van Gelder et al. [64]	→lack of a temporal concordance between the detection of atrial arrhythmias and the occurrence of ischemic stroke in patients with implantable cardiac devices		→ atrial ED which characterizes ACM promotes atrial thrombosis even in the absence of atrial arrhythmias → ACM might be the cause of ESUS (embolic strokes of undetermined source)
	Glotzer et al. [65]			
	Sanna et al. [66]	→ patients with cryptogenic stroke did not present atrial arrhythmias during three years of continuous heart rhythm monitoring after the cerebral event		
	Boeckh-Behren et al. [67]	→ cryptogenic stroke thrombi had a similar composition to cardioembolic thrombi but not to noncardioembolic ones		
	Ivanova et al. [68]	→ AF is associated with atrial endothelial cell desquamation and subendothelial edema in electron microscopy		→ atrial endothelial dysfunction promotes electrical remodeling, leading to AF
	Kato et al. [69]	→ atrial endocardial cells in AF suffer an endothelial–mesenchymal transition, leading to increased collagen production, atrial fibrosis, and remodeling		
	Freestone et al. [70] Freestone et al. [71] Krishnamoorthy et al. [72] Scridon et al. [73]	→ VWF has higher plasma levels in patients with AF compared to patients in SR		→ AF is associated with increased levels of circulating biomarkers of ED: VWF, ADMA
	Black et al. [12]	→ Increased levels of ADMA are associated with AF and predict AF recurrence after cardioversion or ablation		
	Maida et al. [77] Freestone et al.[71] Börschel et al. [78] Komatsu et al. [79] Okawa et al. [80]	→ Patients with AF have lower values of RHI and brachial FMD compared to controls in SR		→ AF is associated with systemic ED
	Kanazawa et al. [84] Yoshino et al. [85] Guazzi et al. [86]	→ Patients with AF cardioversion or ablation had improvements in RHI and FMD after SR restoration		→ AF might contribute per se to ED

MBF, myocardial blood flow; CVR, coronary vascular resistance; PET, positron emission tomography; AF, atrial fibrillation; CAD, coronary artery disease; SR, sinus rhythm; CMR, cardiac magnetic resonance; LA, left atrium; LV, left ventricle; CED, coronary endothelial dysfunction; ED, endothelial dysfunction; ACh, acetylcholine; CMD, coronary microvascular dysfunction; CFR, coronary flow reserve; HFpEF, heart failure with preserved ejection fraction; ACM, atrial cardiomyopathy; FVR, femoral vascular resistance; INOCA, ischemia with nonobstructive coronary arteries; ET-1, endothelin-1; RHI, reactive hyperemia index; FMD, flow mediated dilatation; ESUS, embolic stroke of undetermined source; VWF, Von Willebrand factor; ADMA, asymmetric dimethyl arginine.
